# Uterine Preservation after Vaginal Delivery with Manual Extraction of Focal Placenta Accreta

**DOI:** 10.7759/cureus.6353

**Published:** 2019-12-11

**Authors:** Mary K Marquette, Olga Sarkodie, Anne T Walker, Emily Patterson

**Affiliations:** 1 Obstetrics and Gynecology, East Tennessee State University Quillen College of Medicine, Johnson City, USA; 2 Obstetric and Gynecology, East Tennessee State University Quillen College of Medicine, Johnson City, USA; 3 Pathology, East Tennessee State University Quillen College of Medicine, Johnson City, USA

**Keywords:** placenta, accreta, increta, percreta, uterine, uterus, manual, extraction, trophoblast, vaginal

## Abstract

Placenta accreta spectrum disorder (PASD) is the adherence of the placenta caused by an abnormal trophoblast invasion into the myometrium. It is classified as placenta accreta, placenta increta, and placenta percreta depending on the extent of the invasion. Placenta accreta, defined as the superficial invasion of the placenta to the myometrium, accounts for 75% of PASD. Placenta increta is characterized by chorionic villi invasion deep into the myometrium. Placenta percreta involves placental invasion through the uterus and serosa and into the peritoneal cavity or surrounding viscera. Maternal morbidity and mortality can occur secondary to hemorrhage, disseminated intravascular coagulation, risks associated with blood transfusion, and pelvic and abdominal viscera injury. The standard of care in a known diagnosis of PASD is a cesarean delivery followed by hysterectomy with the placenta in situ. We report a case in which the diagnosis of focal PASD was not known antenatally but suspected after vaginal delivery. The patient subsequently underwent conservative management with uterine preservation and did not require laparotomy.

## Introduction

Placenta accreta spectrum disorder (PASD) is a condition characterized by the abnormal attachment of the placenta to the uterus. It is caused by an abnormal trophoblast invasion into the myometrium [1]. There are three types of PASD, classified based on the extent of the invasion: placenta accreta, placenta increta, and placenta percreta. Placenta accreta is the most common among these, and it is defined as the superficial invasion of the placenta to the myometrium. It accounts for 75% of the PASD cases [1]. Placenta increta occurs when there is a chorionic villi invasion deep into the myometrium. Placenta percreta is characterized by placental invasion through the uterus and serosa and into the peritoneal cavity or surrounding viscera.

## Case presentation

A 34-year-old female patient, gravida two para zero, presented to the labor and delivery unit at Johnson City Medical Center, Johnson City, Tennessee for induction of labor at term. Her medical history was significant for a history of a first-trimester spontaneous abortion managed expectantly, herpes simplex-1, and cervical intraepithelial neoplasia 1. Her prenatal course was complicated by the White's classification A1 gestational diabetes, rubella non-immune status, and a urinary tract infection. The estimated fetal weight at 38 weeks and six days of gestation was 3,680 grams or eight pounds and two ounces, which is at the 73rd percentile for gestational age. Fetal abdominal circumference was at the 91st percentile, while head circumference was only at the 34th percentile. 

At 39 weeks and two days of gestation, the patient was admitted for elective induction of labor. Laboratory evaluation at admission was significant for hemoglobin and hematocrit of 11.4 g/dL and 34.3%, respectively. Induction required cervical ripening with dinoprostone, misoprostol, mechanical dilation, oxytocin, and artificial rupture of membranes. The second stage of labor was complicated by vacuum assistance secondary to maternal exhaustion and resulted in an uncomplicated delivery of a vigorous infant. The infant was born appropriate weight for gestational age. Active management of the third stage of labor was initiated with oxytocin and downward traction on the umbilical cord. 

After thirty minutes of delivery, the placenta failed to deliver and we made the diagnosis of retained placenta. The placenta was manually extracted under transabdominal ultrasound guidance with epidural and intravenous analgesia. After the extraction, an inspection of the placenta showed a thinning in a single area. Re-exploration of the uterine cavity under ultrasound guidance revealed a 2/3-cm placental remnant and a small blood clot, which were extracted manually. The third stage of labor lasted approximately 57 minutes. The total estimated blood loss was 700 ml. The patient had a second-degree perineal laceration, which contributed to total blood loss. After delivery, we palpated the fundus and found it to be firm. We administered broad-spectrum antibiotics as there was a concern about contamination during extraction secondary to semi-sterile conditions of a vaginal delivery. 

**Figure 1:**
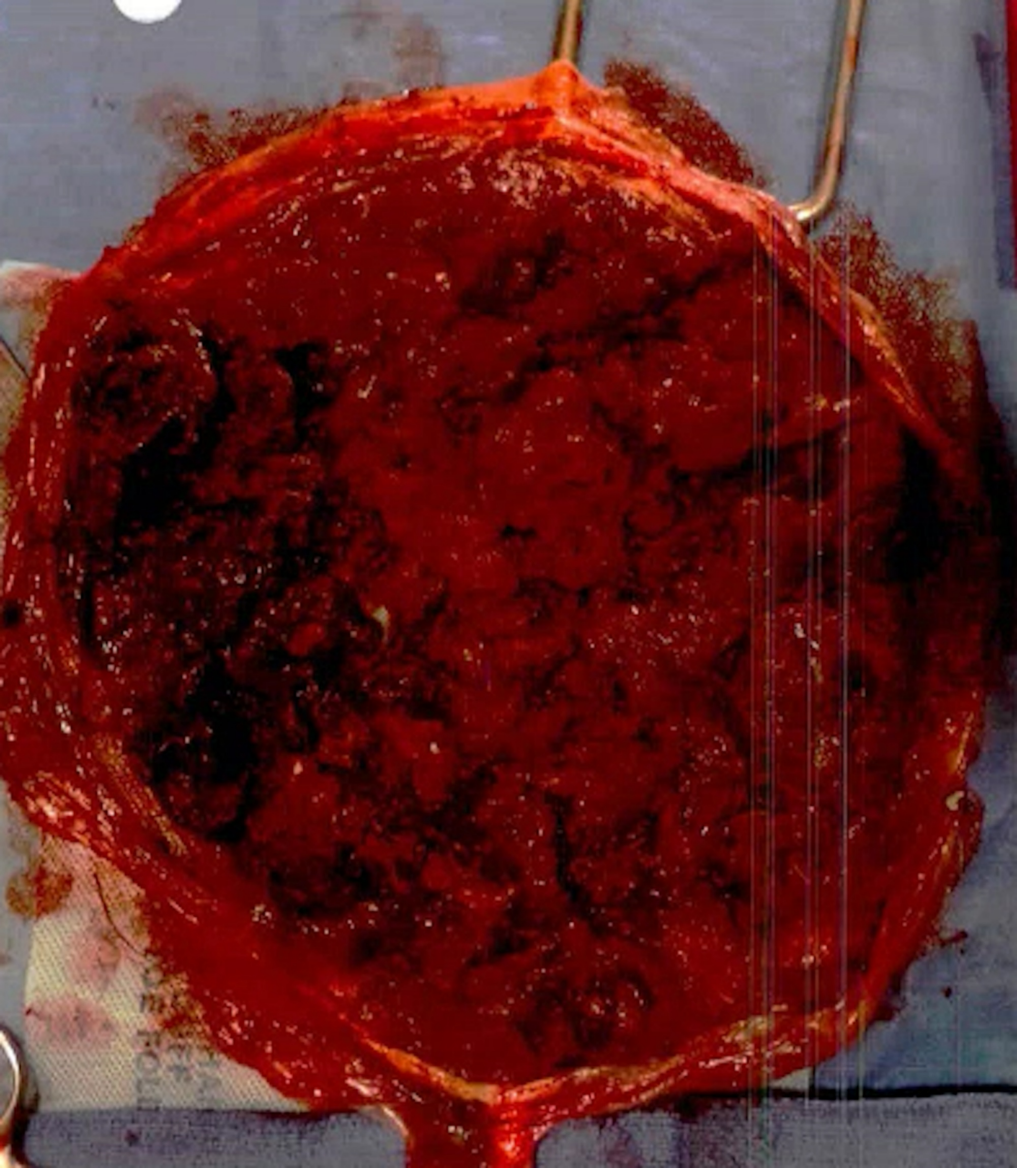
Gross pathology specimen of the maternal side of the placenta after manual extraction

The entire placenta, including non-intact pieces, was sent to pathology for examination (Figure 1). Histologic sections demonstrated decreased- to-focally-absent decidualized myometrium, supporting the clinical impression of placenta accreta. 

In the immediate postpartum period, the patient received one unit of packed red blood cells for symptomatic anemia (hemoglobin and hematocrit: 6.7 g/dL and 22.3%, respectively). She was discharged on postpartum day two. Three days following delivery, the patient was evaluated in our outpatient clinic. She reported appropriate lochia. She received close postpartum follow-up with four postpartum visits within the first six weeks postpartum and one telephone call. She was asked about lochia at each visit. She breastfed without difficulties. The postpartum course was remarkable only for a urinary tract infection, which responded to antibiotics with a negative follow-up urine culture. At the time of preparation of this case report, the patient was still breastfeeding and had not had a return of menses. 

## Discussion

The most common risk factor for PASD is a history of cesarean delivery, with the risk for placenta accreta spectrum increasing as the number of cesarean deliveries increases. Other risk factors include placenta previa, advanced maternal age, prior uterine surgery, prior intrauterine curettage or other intrauterine procedures, multiparity, multifetal gestation, smoking, chronic hypertension, and a history of Asherman syndrome [2-4]. PASD occurs in 2-3% of women diagnosed with placenta previa without a history of cesarean delivery, and placenta previa is present in up to 88% of cases of PASD [3,5-8]. Abnormal results from placental biomarkers are also known risk factors for placenta accreta, but these have not been found to be specific enough for clinical application. 

**Figure 2:**
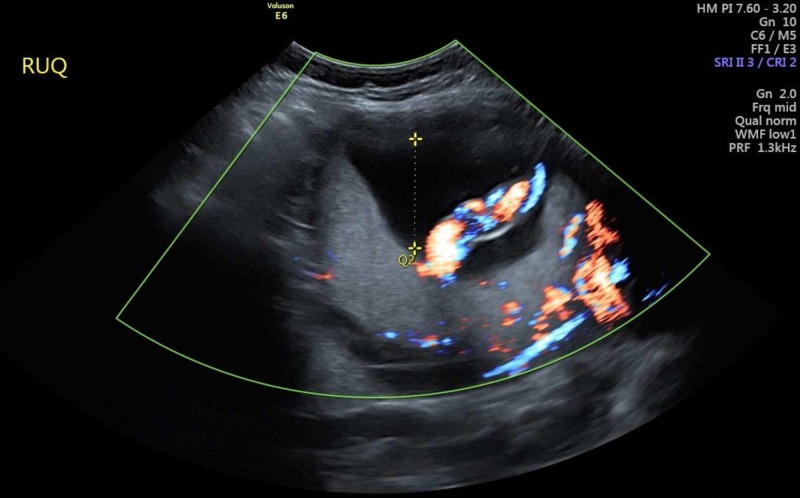
Ultrasound evaluation of the patient demonstrating a lack of evidence of PASD at the fundus

The primary diagnostic modality for antenatal diagnosis is ultrasonography, which has a sensitivity of 77-90%, a positive predictive value of 65-93%, and a negative predictive value of 98% [9-11]. PASD in our patient was not observed on ultrasonography antenatally (Figure 2). 

Though ultrasound features of PASD may be visible as early as the first trimester, most women are not diagnosed until the second or third trimester. Ultrasound features of PASD include multiple vascular lacunae within the placenta, which may be associated with turbulent blood flow, presence of bridging vessels, loss of the normal hypoechoic zone (“clear zone”) between the placenta and the myometrium, subplacental hypervascularity, decreased retroplacental myometrial thickness (less than 1 mm), gaps in myometrial blood flow, and abnormalities of the uterine serosa-bladder interface [3,10-13]. Features that should raise suspicion for PASD are the presence of an anterior low-lying placenta or a placenta previa in a woman with a history of cesarean delivery or other uterine surgery [3,10]. Of note, abnormalities associated with PASD are common in pregnancies not complicated by PASD as well [1]. MRI is a useful adjunct used for diagnosis and perioperative planning. The optimal timing of ultrasound assessment is unclear [1]. 

Antenatal diagnosis of placenta accreta is preferable as outcomes are optimized with delivery at a level-III/IV maternal-care facility by a comprehensive multidisciplinary care team [6-8,14]. The gold standard for the management of placenta accreta is cesarean delivery followed by total or subtotal hysterectomy with the placenta left in situ after the delivery of the fetus. Attempts at placenta removal have been associated with an increased risk of maternal morbidity and mortality secondary to hemorrhage [3,15]. Conservative management of placenta accreta is aimed at uterine preservation and should be considered on a case-by-case basis. Conservative management may be successful for patients with focal placental adherence [16]. This involves removal of the placenta by either manual extraction or surgical excision followed by the repair of the resulting defect or wedge resection of the uteroplacental defect followed by uterine closure, typically in two layers [1]. An alternative to placental removal is expectant management, in which the placenta is either partially or totally left in situ with the expectation of autolysis, or intrauterine tamponade [3,17-18]. Conservative or expectant management may be followed by methotrexate administration and/or pelvic devascularization [3]. In our review of the medical literature, there was a paucity of information on focal placenta accreta managed without laparotomy and hysterotomy. 

## Conclusions

The gold standard for the management of PASD is cesarean delivery followed by a hysterectomy, which is associated with lower maternal morbidity and mortality. However, in the case of a focal placenta accreta diagnosed at the time of delivery, there may be a role for conservative management in selected patients who desire uterine preservation and want to avoid laparotomy. A patient who has a vaginal delivery complicated by suspected focal placenta accreta may opt for a manual extraction or curettage to remove the adherent placenta. This preserves fertility and avoids the morbidity associated with laparotomy. Important considerations include a discussion of the risks involved, adequate anesthesia, the use of ultrasound guidance, blood product availability, and patient reliability for follow-up. Close postpartum follow-up with an evaluation of vaginal bleeding is essential, as an increase in vaginal bleeding may warrant repeat ultrasound assessment or quantitative beta-human chorionic gonadotropin measurement. If the patient is planning to breastfeed, this should be discussed at each visit as well, since retained placental tissue may cause delayed or absent lactogenesis. A case series on the conservative management of PASD would provide more information on risks and benefits in these patients, as well as complications that can occur.

## Appendices

Declaration of Patient Consent 

The authors have obtained all appropriate patient consent, including consent to use images and other clinical information. The patient understands that there is no guarantee of anonymity.
